# The Clinical Importance of *Campylobacter concisus* and Other Human Hosted *Campylobacter* Species

**DOI:** 10.3389/fcimb.2018.00243

**Published:** 2018-07-24

**Authors:** Fang Liu, Rena Ma, Yiming Wang, Li Zhang

**Affiliations:** School of Biotechnology and Biomolecular Sciences, University of New South Wales, Sydney, NSW, Australia

**Keywords:** *Campylobacter concisus*, inflammatory bowel disease, human hosted *Campylobacter*, virulence factor, bacterial marker, transmission, oral *Campylobacter*, pathogenic mechanism

## Abstract

Historically, Campylobacteriosis has been considered to be zoonotic; the *Campylobacter* species that cause human acute intestinal disease such as *Campylobacter jejuni* and *Campylobacter coli* originate from animals. Over the past decade, studies on human hosted *Campylobacter* species strongly suggest that *Campylobacter concisus* plays a role in the development of inflammatory bowel disease (IBD). *C. concisus* primarily colonizes the human oral cavity and some strains can be translocated to the intestinal tract. Genome analysis of *C. concisus* strains isolated from saliva samples has identified a bacterial marker that is associated with active Crohn's disease (one major form of IBD). In addition to *C. concisus*, humans are also colonized by a number of other *Campylobacter* species, most of which are in the oral cavity. Here we review the most recent advancements on *C. concisus* and other human hosted *Campylobacter* species including their clinical relevance, transmission, virulence factors, disease associated genes, interactions with the human immune system and pathogenic mechanisms.

## Introduction

*Campylobacter*, along with *Arcobacter* and *Sulfurospirillum*, are the three genera that belong to the family, Campylobacteraceae. *Campylobacter* species are Gram-negative, curved or spiral shaped, and most of them are motile with a single polar flagellum present at one or both ends of the bacteria, allowing them to have a corkscrew-like motion during movement (Lastovica et al., 2014). *Campylobacter* species have low G + C content in their genome, and the median G + C content for most of the *Campylobacter* species ranges from 28 to 40% (Pruitt et al., 2007). There are few *Campylobacter* species which have G + C content of more than 40% in their genomes, including *Campylobacter curvus, Campylobacter rectus, Campylobacter showae* and *Campylobacter gracilis* (Pruitt et al., 2007). A majority of the *Campylobacter* species are microaerophiles, while some require anaerobic conditions for their growth (Debruyne et al., 2008).

Most *Campylobacter* species live as normal flora in the gastrointestinal tract of various animals (Lastovica et al., 2014). Some of these animal hosted *Campylobacter* species, such as *Campylobacter jejuni* and *Campylobacter coli*, can cause acute bacterial gastroenteritis in humans through consumption of contaminated food or water (Galanis, 2007). In addition to gastroenteritis, *C. jejuni* also causes Guillain-Barré syndrome, due to molecular mimicry between its sialylated lipooligosaccharides and the human nerve gangliosides (Takahashi et al., 2005).

As *C. jejuni* and *C. coli* are the main *Campylobacter* pathogens which cause human acute intestinal disease and they originate from animal sources, Campylobacteriosis has historically been considered to be zoonotic. Several *Campylobacter* species utilize humans as their natural host and accumulated evidence supports their role in chronic inflammatory diseases of the human intestinal tract. Here we review recent advancements on human hosted *Campylobacter* species, their clinical relevance, transmission, virulence factors, disease associated genes, interactions with human immune system and pathogenic mechanisms. Most of the studies on the human hosted *Campylobacter* species in the past decade were on *Campylobacter concisus*, this bacterium is therefore the focus of this review. In addition, other human hosted *Campylobacter* species were also reviewed.

## The natural hosts of *Campylobacter* species and human diseases associated with *Campylobacter* species

The natural host of a bacterium refers to the host that the bacterium normally lives and reproduces (Haydon et al., 2002; Control and Prevention, 2006). Bacterial species are usually not harmful to their hosts, although there are exceptions. For example, *Helicobacter pylori* is a human hosted bacterial species, causing gastritis and gastric ulcers and being a risk factor for gastric cancer (Roesler et al., 2014).

**Figure 1:**
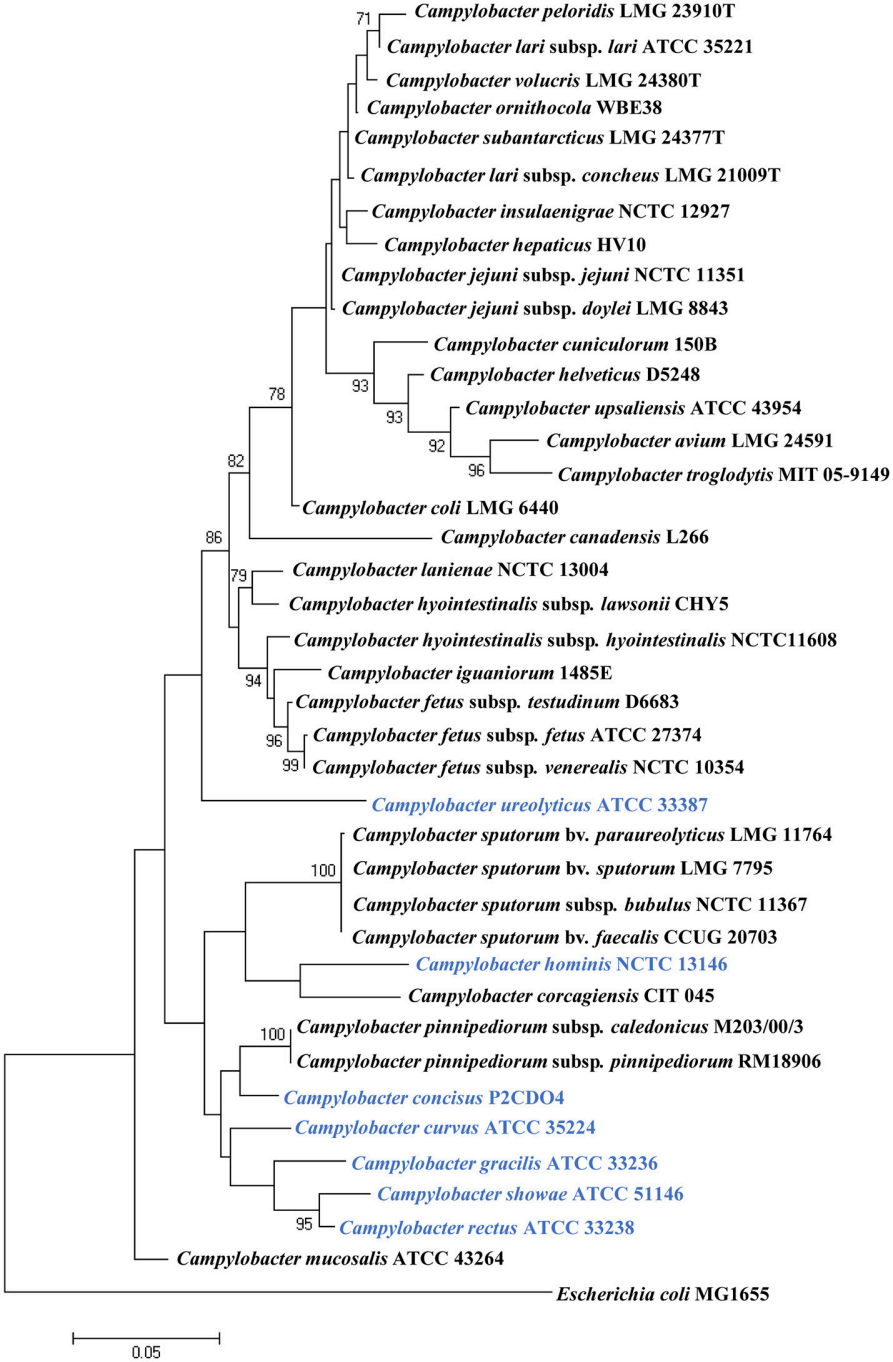
Phylogenetic tree based on the 16S rRNA gene of *Campylobcter* species. The tree was generated using the maximum likelihood method implemented in MEGA7. *Bootstrap* values were generated from 1000 replicates. *Bootstrap* values of more than 70 were indicated. *Escherichia coli* MG1655 was included as an outgroup. Human hosted *Campylobacter* species are in blue. *Campylobacter geochelonis* was not included because its 16S rRNA sequence was not available.

To date, 40 *Campylobacter* species and subspecies have been isolated from a wide variety of animal or human sources (Figure 1). Many *Campylobacter* species are naturally hosted by domesticated animals raised as food such as chicken, cattle and pigs (Lastovica et al., 2014). They survive as commensal bacteria in their hosts, and some species, such as *C. jejuni* and *C. coli*, can cause human diseases. The main human disease caused by animal hosted pathogenic *Campylobacter* species is acute gastroenteritis and clinical disorders can also arise if bacterial species colonizing sterile sites of the body (Table 1). A number of *Campylobacter* species are also able to cause diseases in animals. For example, *Campylobacter fetus* is known to cause abortion in bovine and ovine, and *Campylobacter hepaticus* is the causative agent of spotty liver disease in chicken (Campero et al., 2005; Van et al., 2016).

**Table 1:** Clinical relevance of animal hosted *Campylobacter* species.

***Campylobacter* species**	**Isolation sources**	**Clinical relevance of human diseases**	**References**
*Campylobacter coli*	Gastroenteritis: feces Bacteraemia: blood Septic abortion: blood; maternal placenta; amniotic fluid Acute cholecystitis: gallbladder Retroperitoneal abscess Meningitis: CSF	Gastroenteritis^*^ Abortion^*^ Bacteraemia^*^	Skirrow, 1977; Kist et al., 1984; Blaser et al., 1986; Møller Nielsen et al., 1997; Lastovica and Roux, 2000; Galanis, 2007; Liu Y. H. et al., 2017;
*Campylobacter fetus* subsp. *fetus*	Bacteraemia: blood Gastroenteritis: feces Meningitis: feces; blood; CSF Chorioamnionitis: blood Cellulitis lesion: subcutaneous aspirate Cellulitis and bacteraemia: blood; feces Abortion: vagina; feces; blood; gastric aspirate; skin; liver; spleen; lung; spinal fluid Postsurgical abscess: groin abscess Post abortion infection: amniotic fluid Hemiparesis and aphasia: blood Cystic fibrosis: feces Surgical fever: blood Fever, chills, endocarditis: blood Immune deficiency disease: blood Sepsis, encephalitis, fever, myalgia: blood and CSF Cellulitis and diarrhea: ankle abscess Post-neurosurgery for metastatic esophagocardial carcinoma: brain abscess Chronic alcoholism: brain abscess Prematurity: brain abscess Heroin and barbiturate abuse: pulmonary abscess Alcoholism: gluteal abscess; blood Post-hemilaminectomy for disc herniation: epidural mass in pyogenic vertebral osteomyelitis Hyperthyroidism: thyroid gland abscess	Bacteraemia^*^ Abortion^*^ Meningitis^*^ Abscess^*^ Gastroenteritis	Blaser et al., 1980a,b; Edmonds et al., 1985; Francioli et al., 1985; La Scolea, 1985; Klein et al., 1986; Simor et al., 1986; Morrison et al., 1990; Sauerwein et al., 1993; Kwon et al., 1994; Neuzil et al., 1994; Steinkraus and Wright, 1994; Morooka et al., 1996; Ichiyama et al., 1998; Lastovica and Roux, 2000; Viejo et al., 2001; Krause et al., 2002; Fujihara et al., 2006; De Vries et al., 2008; Liu Y. H. et al., 2017
	Health status unknown: Blood and synovial fluid		
*Campylobacter fetus* subsp. *testudinum*	Leukemia: blood Liver cancer (bloody diarrhea, pulmonary edema): pleural fluid Asthma: hematoma Lymphoma, hypertension, and heart disease (fever, chills, rigor, cough, and diarrhea): blood Diarrhea: bile Diabetes (cellulitis of leg): blood	Leukemia^*^	Tu et al., 2004; Patrick et al., 2013
*Campylobacter fetus* subsp. *venerealis*	Bacteraemia: blood Infective aneurysm: blood Vaginosis		Garcia et al., 1995; Tu et al., 2001; Hagiya et al., 2015; Liu Y. H. et al., 2017
	Health status unknown: blood		
*Campylobacter helveticus*	Health status unknown: feces		Lawson et al., 1997
*Campylobacter hyointestinalis (*subsp. *hyointestinalis* and *lawsonii)*	Proctitis: rectum Gastroenteritis: feces	Gastroenteritis^*^	Fennell et al., 1986; Edmonds et al., 1987; Lastovica and Roux, 2000; Gorkiewicz et al., 2002
*Campylobacter insulaenigrae*	Gastroenteritis: blood Bacteraemia: blood	Gastroenteritis Bacteraemia^*^	Chua et al., 2007
*Campylobacter jejuni (*subsp. *jejuni* and *doylei)*	Bacteraemia: blood; feces Gastroenteritis: feces Sepsis: blood, feces, placenta Meningitis: CSF Appendicitis: appendix Myocarditis: feces Reactive arthritis: feces Guillain-Barré syndrome: feces Fisher syndromes: feces; gastric biopsy; CSF Recurrent colitis: blood; feces Acute cholecystitis: gallbladder Urinary tract infection: urine Chronic renal failure: peritoneal prodialysis fluid Ovarian cyst: peritoneal cyst fluid Thoracic wall abscess: thoracic wall Meningitis and hypogammaglobulinemia: CSF	Gastroenteritis^*^ Bacteraemia^*^ Guillain-Barré syndrome^*^ Meningitis^*^ Appendicitis Myocarditis Reactive arthritis	Skirrow, 1977; Thomas et al., 1980; Gilbert et al., 1981; Megraud et al., 1982; Chan et al., 1983; Blaser et al., 1986; Dhawan et al., 1986; Goossens et al., 1986; Klein et al., 1986; Kohler et al., 1988; Korman et al., 1997; Meyer et al., 1997; Møller Nielsen et al., 1997; Manfredi et al., 1999; Lastovica and Roux, 2000; Wolfs et al., 2001; Hannu et al., 2002, 2004; Cunningham and Lee, 2003; Takahashi et al., 2005; Lastovica, 2006; Galanis, 2007; Pena and Fishbein, 2007; Mortensen et al., 2009; Liu Y. H. et al., 2017
*Campylobacter lanienae*	Healthy: feces		Logan et al., 2000
*Campylobacter lari (*subsp. *concheus* and *lari)*	Urinary tract infection: urine Bacteraemia: blood Gastroenteritis: feces	Bacteraemia^*^	Bézian et al., 1990; Morris et al., 1998; Lastovica and Roux, 2000; Martinot et al., 2001; Krause et al., 2002; Werno et al., 2002
*Campylobacter mucosalis*	Gastroenteritis: feces		Figura et al., 1993
*Campylobacter peloridis*	Health status unknown: feces		Debruyne et al., 2009
*Campylobacter sputorum (biovar faecalis, paraureolyticus, sputorum*, and *bubulus)*	Gastroenteritis: feces Axillary abscess Leg abscess Pus from pressure sore	Gastroenteritis Abscess^*^	Roop Ii et al., 1985; Steele et al., 1985; Lindblom et al., 1995; Tee et al., 1998; De Vries et al., 2008
	Health status unknown: oral cavity; feces		
*Campylobacter troglodytis*	Gastroenteritis: feces		Platts-Mills et al., 2014
*Campylobacter upsaliensis*	Abortion: blood and fetoplacental material Bacteraemia: blood Gastroenteritis: feces Breast abscess	Gastroenteritis Bacteraemia^*^	Lastovica et al., 1989; Gurgan and Diker, 1994; Jimenez et al., 1999; Lastovica and Roux, 2000; Lastovica and Le Roux, 2001; De Vries et al., 2008

*CSF, cerebrospinal fluid*.

* *Campylobacter species that have established associations with human diseases or have been isolated from a sterile site. Species which have not yet been isolated from humans were not included*.

*Campylobacter* species hosted by humans include *C. concisus, C. curvus, C. gracilis, Campylobacter hominis, C. rectus*, and *C. showae*; to date these six *Campylobacter* species have only been isolated from humans (Hariharan et al., 1994; Zhang, 2015). *Campylobacter ureolyticus* was isolated from human samples in most cases, with only one study isolating *C. ureolyticus* from the endometria of healthy horses (Hariharan et al., 1994). Therefore, in this review, *C. ureolyticus* is considered as a human hosted *Campylobacter* species. In contrast to animal hosted *Campylobacter* pathogens, the human hosted *Campylobacter* pathogens are more often involved in chronic inflammatory conditions of the gastrointestinal tract (Table 2).

**Table 2:** Clinical relevance of human hosted *Campylobacter* species.

***Campylobacter* species**	**Isolation sites in human**	**Clinical relevance of human diseases**	**References**
*Campylobacter concisus*	Healthy: saliva; subgingival site; intestinal biopsy; feces	Inflammatory bowel disease^*^ Diarrheal diseases Barrett's esophagus^*^ Periodontal disease	Tanner et al., 1981; Lindblom et al., 1995; Lastovica and Roux, 2000; Macuch and Tanner, 2000; Lastovica, 2006; Macfarlane et al., 2007; De Vries et al., 2008; Zhang et al., 2010; Kalischuk and Inglis, 2011; Mukhopadhya et al., 2011; Nielsen et al., 2011, 2013; Blackett et al., 2013; Mahendran et al., 2013; Zhang, 2015; Kirk et al., 2016
	IBD: saliva; intestinal biopsy Diarrhea: feces Barrett's esophagus: esophageal aspirate; distal esophageal biopsy Brain abscess: secondary to chronic frontal osteomyelitis Periodontal disease: subgingival site		
*Campylobacter curvus*	Healthy: subgingival site	Gastroenteritis Abscess	Koga et al., 1999; Lastovica and Roux, 2000; Macuch and Tanner, 2000; Abbott et al., 2005; Petersen et al., 2007; De Vries et al., 2008; Mendz et al., 2014; Horio et al., 2017
	Periodontal disease: subgingival and periodontitis site Thoracic empyema: pleural effusion Premature birth: vaginal swabs Alveolar abscess Metastatic ovarian cancer: liver abscess Liver abscess: blood Lung cancer: bronchial abscess Guillain-Barré syndrome: feces Fisher's syndrome: feces Gastroenteritis: feces		
*Campylobacter gracilis*	Healthy: subgingival site	Periodontal disease^*^	Tanner et al., 1981; Johnson et al., 1985; Yu and Chen, 1997; Macuch and Tanner, 2000; De Vries et al., 2008; Shinha, 2015
	Bacteraemia: blood Brain abscess (post-partum) Tubo-ovarian abscess Periodontal disease: subgingival and periodontitis site Visceral or head and neck infection		
*Campylobacter hominis*	Healthy: feces	Septicaemia	Lawson et al., 1998, 2001; Linscott et al., 2005; Zhang et al., 2009
	Septicaemia: blood CD: intestinal biopsy		
*Campylobacter rectus*	Healthy: subgingival site	Periodontal diseases^*^ IBD	Von Troil-Lindén et al., 1995; Lastovica and Roux, 2000; Macuch and Tanner, 2000; Han et al., 2005; Macfarlane et al., 2007; De Vries et al., 2008; Mahlen and Clarridge, 2009; Zhang et al., 2009; Man et al., 2010b; López et al., 2011; Mukhopadhya et al., 2011; Lee et al., 2012; Leo and Bolger, 2014; Noël et al., 2018
	Periodontal disease: subgingival and periodontitis site Barrett's esophagus: distal esophageal mucosal biopsy Fatal thoracic empyema: pleural liquid Septic cavernous sinus thrombosis: blood Gastroesophageal adenocarcinoma: palate abscess Breast abscess Vertebral abscess Gastroenteritis: feces		
*Campylobacter showae*	Healthy: subgingival site; gingival crevices	IBD	Etoh et al., 1993; Macuch and Tanner, 2000; De Vries et al., 2008; Zhang et al., 2009; Man et al., 2010b; Suzuki et al., 2013
	Periodontal disease: subgingival and periodontitis site CD: intestinal biopsy Intraorbital abscess Bacteraemia: blood		
*Campylobacter ureolyticus*	Healthy: Male: urine Female: genital tract	IBD Gastroenteritis Genital tract diseae	Duerden et al., 1982, 1987, 1989; Johnson et al., 1985; Bennett et al., 1990, 1991; Petersen et al., 2007; Zhang et al., 2009; Bullman et al., 2011; Mukhopadhya et al., 2011; O'doherty et al., 2014
	CD: intestinal biopsy Gastroenteritis: feces Periodontal disease: deep periodontal pockets Superficial soft tissue or bone infections Male: Non-gonococcal, non-chlamydial urethritis Non-gonococcal urethritis Superficial necrotic or gangrenous lesions Penile wound Female: Perineal, genital and peripheral ulcers Genital tract: excess vaginal discharge; lower genital tract symptoms		
	Health status unavailable: Amniotic fluid; urine		

* *Campylobacter species that have established associations with human diseases or have been isolated from a sterile site*.

## C. concisus

*C. concisus* has a curved or spiral shape and a single polar flagellum, with the size being 0.5–1 by 4 μm (Tanner et al., 1981). Its colony appears as convex shaped, translucent and is ~1 mm in diameter (Tanner et al., 1981). In early literature, *C. concisus* was described as a microaerophile, due to the growth of this bacterium under microaerophilic atmosphere enriched with hydrogen (H_2_) (Lastovica, 2006). Later, Lee et al. demonstrated that *C. concisus* is not a microaerophile, as they found that none of the 57 *C. concisus* strains grew in the microaerophilic conditions generated using the Oxoid BR56A and CN25A gas-generation systems (Lee et al., 2014). These *C. concisus* strains were able to grow under anaerobic conditions, with tiny colonies observed after 3 days of culture under anaerobic conditions, showing that *C. concisus* is an anaerobic bacterium. The anaerobic condition used in the study was generated using AN25A gas-generation system which absorbs oxygen with simultaneous production of CO_2_.

*C. concisus* is a chemolithotrophic bacterium, capable of using H_2_ as a source of energy to markedly increases its growth (Lee et al., 2014). *C. concisus* is able to oxidize H_2_ under both anaerobic and microaerobic conditions, although greater growth was observed under anaerobic conditions in the presence of 2.5–10% H_2_ (Lee et al., 2014). *C. concisus* is catalase negative, contributing to its inability to grow under microaerobic conditions (Tanner et al., 1981).

### Transmission of *C. concisus*

**Figure 2:**
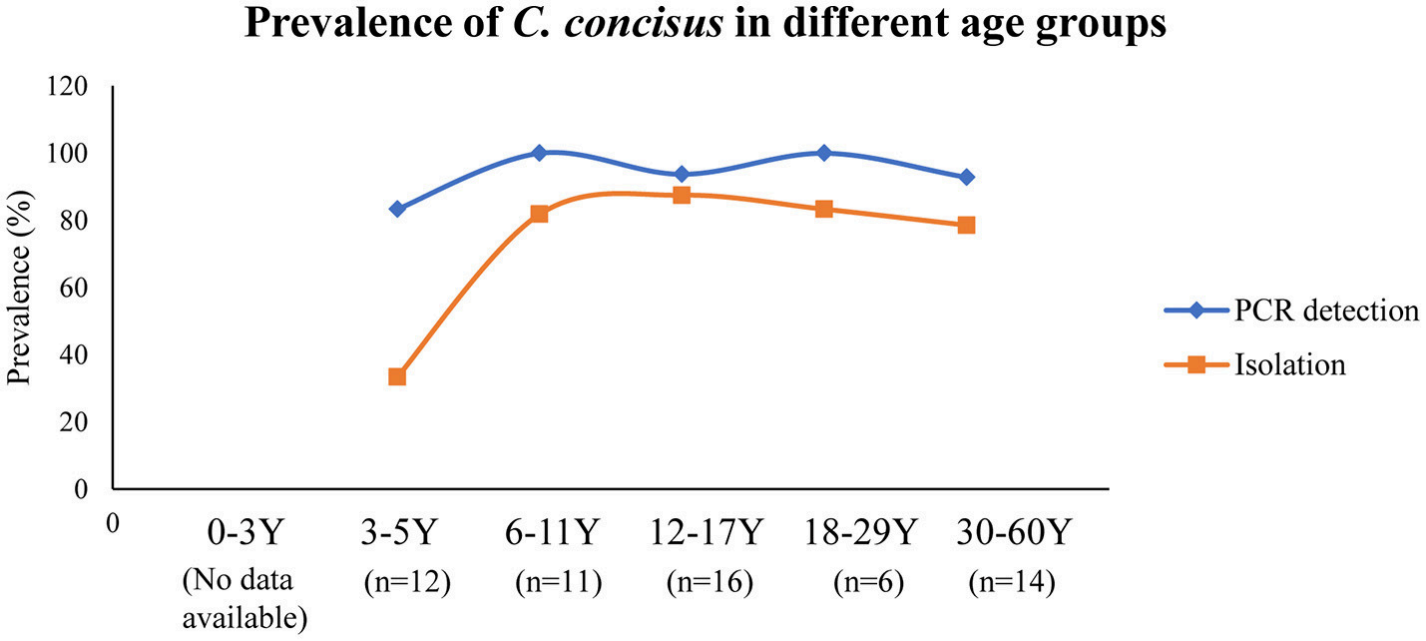
Detection and isolation rates of *C. concisus* from saliva samples in different age groups. The PCR detection rate of *C. concisus* from children of different age groups varied between 80 and 100%. However, the isolation rate of *C. concisus* from children at 3–5 years was only 33% (4/12), which was significantly lower than that in other age groups, indicating younger children were colonized with lower numbers of bacteria. Data are from Zhang et al. (2010).

Currently, humans are the only known hosts of *C. concisus* with oral cavity being its natural colonization site (Zhang et al., 2010; Mahendran et al., 2013). *C. concisus* has been isolated from saliva samples of children as early as 3 years old, although the positive isolation rate was significantly lower than the other age groups (33 vs. 79–88%); and the highest *C. concisus* isolation rate was seen in the age group of 12–17 (Zhang et al., 2010) (Figure 2). When a PCR method was used for detection, the children from the 3 to 5 years old age bracket had a detection rate that was similar to other age groups (83 vs. 93–100%). These data show that although *C. concisus* colonize humans at the early stages of life, 3–5 year old children have lower bacterial loads of *C. concisus* in their saliva as compared to older children and the adults. Currently, no data are available regarding *C. concisus* colonization in children below 3 years old.

Given that *C. concisus* colonizes the oral cavity, transmission would occur through saliva. The stability of *C. concisus* in saliva samples is related to sample storage. We were able to isolate *C. concisus* from saliva samples stored at 4°C for 3–6 days. However, we were unable to isolate *C. concisus* from the same saliva samples after storage at room temperature for 24 h. This suggests that in addition to direct contact, *C. concisus* contaminated food or drinks, particularly those stored in refrigerators, may also play a role in *C. concisus* transmission. Multiple strains of *C. concisus* have been isolated from saliva samples and enteric samples of given individuals, suggesting a possible dynamic colonization of new *C. concicus* strains in the human gastrointestinal tract (Ismail et al., 2012).

### Association of *C. concisus* with human diseases

#### Gingivitis and periodontitis

Gingivitis is a common bacterial disease which affects 90% of the population (Coventry et al., 2000). The oral mucosa consists of stratified squamous epithelial cells (Lumerman et al., 1995). Periodontitis develops when gingivitis is not well-treated (Pihlstrom et al., 2005). As disease progresses, a loss of attachment between the gingivae and the teeth leads to the formation of a periodontal pocket, which then allows extensive colonization by anaerobic bacteria causing further inflammation of the mucosa (Highfield, 2009). In periodontitis patients, higher proportions of Gram-negative and anaerobic bacterial species are present in the oral cavity as compared to healthy controls (Newman and Socransky, 1977). *C. concisus* was initially isolated from the oral cavity of patients with gingivitis and periodontitis (Tanner et al., 1981). However, studies have not revealed a clear association between *C. concisus* and gingivitis and periodontitis, its role in human oral inflammatory diseases remains unclear.

#### Barrett's esophagus

The esophagus is a muscular conduit with stratified squamous epithelial layers; its mucosa is colonized by microbes dominated by members of the genus *Streptococcus* (Di Pilato et al., 2016). Disturbances of the microbiota composition, such as the abnormal enrichment of some Gram-negative bacteria including *Campylobacter* spp. has been reported to be associated with gastroesophageal reflux disease (GERD), and is suggested to contribute to the development toward Barrett's esophagus and oesophageal adenocarcinoma (Di Pilato et al., 2016).

By analyzing the microbiome composition in biopsy samples of the distal esophagus collected from normal individuals and patients with esophagitis or Barrett's esophagus, Yang et al. found that type I microbiome (Gram-positive aerobic microbiome) was mainly associated with normal esophagus (11/12, 91.7%), while type II microbiome (Gram-negative anaerobic microbiome) was more closely associated with abnormal esophagus including esophagitis and Barrett's esophagus (13/22, 59.1%) (Yang et al., 2009). Furthermore, *Campylobacter* species was found to be one of the genera with increased abundance in type II microbiome (Yang et al., 2009).

By using bacterial cultivation methods, Macfarlane et al. found that high levels of *C. concisus* and *C. rectus* were present in oesophageal aspirate and mucosal samples of patients with Barrett's esophagus, but not in control subjects (Macfarlane et al., 2007). Similar findings were reported by another study, in which the *Campylobacter* genus dominated by *C. concisus* colonized patients with GERD and Barrett's esophagus with increased bacterial counts, accompanied by a significant decrease in bacterial counts for all other genera (Blackett et al., 2013). This relationship was not observed in patients with oesophageal adenocarcinoma. It is possible that *C. concisus* contributes to the inflammation associated with GERD and Barrett's esophagus.

#### Gastroenteritis

Gastroenteritis is an inflammatory condition of the gastrointestinal tract which is characterized by diarrhea, abdominal pain, fever and vomiting (Galanis, 2007). It is usually self-limiting within 2–5 days (Galanis, 2007). Gastroenteritis can be caused by bacteria, viruses, parasites and fungi; the most common causes are rotavirus and bacterial species such as *Escherichia coli, C. jejuni*, and *C. coli* (Galanis, 2007).

A number of studies have reported the isolation of *Campylobacter* species other than *C. jejuni* and *C. coli* in diarrheal stool samples. Lindblom et al. found that in stool samples from diarrheal patients, *Campylobacter upsaliensis, Campylobacter sputorum*, and *C. concisus* were the most common species in addition to *C. jejuni*, of which *C. concisus* was only isolated from children (Lindblom et al., 1995). Similarly, Lastovica et al. reported that in addition to *C. jejuni, C. concisus* was the second most frequently isolated *Campylobacter* species from diarrheic stools of pediatric patients (Lastovica and Roux, 2000). Nielsen et al. also found that *C. concisus* was more frequently found in diarrheal stool samples of young children and elderly (Nielsen et al., 2013). The same group later reported a clinical study comparing clinical manifestations between adult patients infected with *C. concisus* and *C. jejuni/C. coli*, and they showed that although *C. concisus* infection seems to induce a milder course of acute gastroenteritis in comparison to that induced by *C. jejuni/C. coli*, it is associated with prolonged diarrhea (Nielsen et al., 2012). Recently, Serichantalergs et al. reported a significantly higher detection rate of *C. concisus* in traveller's diarrhea cases as compared to that in asymptomatic controls in Nepal (Serichantalergs et al., 2017). Another recent study by Tilmanne et al. showed that *C. concisus* had a similar prevalence in children with acute gastroenteritis as compared with the control group (Tilmanne et al., 2018). Unfortunately, most of the studies that reported the isolation of *C. concisus* from diarrheal stool samples did not have control fecal samples from healthy individuals, making it difficult to judge the role of this bacterium in gastroenteritis; and for those studies with control groups included, the results were controversial. Thus, whether *C. concisus* plays a role in gastroenteritis remains to be investigated.

#### Inflammatory bowel disease

Inflammatory bowel disease (IBD) is a chronic inflammatory disease of the gastrointestinal tract with Crohn's disease (CD) and ulcerative colitis (UC) being two of its major forms (De Souza and Fiocchi, 2016). The two forms of IBD mainly differ in pathology. CD is characterized by having discontinuous “skip lesions” of transmural inflammation and the abnormalities can be found throughout the gastrointestinal tract (Walsh et al., 2011). In contrast to CD, the inflammation involvement for UC is usually confined to the mucosa and submucosa without skip lesions and mostly occurs in the large intestine (Walsh et al., 2011).

The etiology of IBD is not fully understood. Accumulated evidence has suggested that the mucosal immune system in genetically predisposed individuals has mounted responses to intestinal commensal bacterial species, which is believed to contribute to the pathogenesis of IBD (Knights et al., 2013). Intestinal commensal bacterial species have co-evolved with the intestinal mucosal immune system thus the immune responses to intestinal commensal bacterial species would need an external trigger. Microbes that have the ability to cause a prolonged primary intestinal barrier defect or alter the mucosal immune system are more likely to trigger IBD.

*C. concisus* is commonly present in the oral cavities of almost all individuals. However, patients with IBD have a significantly higher prevalence of *C. concisus* detected in their intestinal tissues as compared to healthy controls (Zhang et al., 2009, 2014; Man et al., 2010b; Mahendran et al., 2011; Mukhopadhya et al., 2011; Kirk et al., 2016). Comparison of the housekeeping genes and genomes of oral and enteric *C. concisus* strains suggests that enteric strains originate from oral *C. concisus* strains (Ismail et al., 2012; Chung et al., 2016).

*C. concisus, C. hominis, C. showae*, and *C. ureolyticus* have also been isolated from patients with CD. Additionally, *C. homonis, C. showae, C. rectus, C. gracilis*, and *C. ureolyticus* have been detected from fecal specimens of children with newly diagnosed CD (Zhang et al., 2009; Man et al., 2010b). However, no association has been found between the prevalence of these *Campylobacter* species in CD patients and healthy controls.

### Genomospecies of *C. concisus*

*C. concisus* strains can be separated into two major genomospecies (GS), consistently defined by the analysis of core genomes and housekeeping genes (Istivan, 2005; Miller et al., 2012; Mahendran et al., 2015; Chung et al., 2016; Nielsen et al., 2016). *C. concisus* 23S rRNA gene has polymorphisms, which was also used to define the genomospecies by comparison of the entire 23S rRNA gene sequence or PCR amplification of 23S rRNA gene fragments (Engberg et al., 2005; Kalischuk and Inglis, 2011; On et al., 2013; Huq et al., 2017; Wang et al., 2017).

Quantitative PCR methods targeting the polymorphisms of the 23S rRNA gene revealed that there were more GS2 than GS1 *C. concisus* in samples collected from the upper and lower gastrointestinal tract of both patients with IBD and healthy controls, suggesting that GS2 *C. concisus* is better adapted to the human gastrointestinal tract (Wang et al., 2017). A meta-analysis of the composition of the isolated GS1 and GS2 *C. concisus* strains showed similar findings except that in healthy individuals, a significantly lower number of GS2 *C. concisus* strains than GS1 *C. concisus* were isolated from fecal samples. This suggests a potential difference in the *C. concisus* strains or the enteric environment between patients with gastrointestinal diseases and healthy controls (Wang et al., 2017).

The two GS of *C. concisus* do not differ in morphology but have GS-specific genes and may have different pathogenic potentials. By examining *C. concisus* strains isolated from diarrheal fecal samples, Engberg et al. found that bloody diarrhea was only present in individuals infected with GS2 *C. concisus* strains (Engberg et al., 2005). Furthermore, Kalischuk and Inglis demonstrated that GS2 *C. concisus* exhibited higher levels of epithelial invasion and translocation (Kalischuk and Inglis, 2011). Similarly, Ismail et al. reported that the oral *C. concisus* strains that were more invasive to intestinal epithelial cells were GS2 strains (Ismail et al., 2012).

Recently, comparative genomic analyses have identified two novel genomic islands CON_PiiA and CON_PiiB that carry proteins homologous to the type IV secretion system, LepB-like and CagA-like effectors (Chung et al., 2016). CON_PiiA and CON_PiiB were found in strains from both GS1 and GS2 (Chung et al., 2016). The effects of the proteins possessed by CON_PiiA and CON_PiiB on human cells require further examination.

Both GS1 and GS2 contain diverse *C. concisus* strains. The isolation sources of *C. concisus* strains do not seem to contribute to the phylogenetic relatedness. *C. concisus* strains isolated from saliva, intestinal biopsies and feces are found in both GS1 and GS2, as are the strains isolated from patients with enteric disease and healthy controls. A recent study examining the genomes of 104 *C. concisus* strains isolated from saliva, mucosal biopsies and fecal samples of patients with IBD, gastroenteritis and healthy individuals showed that sampling site rather than disease phenotype was associated with the particular GS (Kirk et al., 2018). In that study, authors reported that genes involved in cell membrane synthesis were common in oral strains, while those related to cell transport, metabolism, and secretory pathways were more often found in enteric isolates. These results indicate that GS alone is unable to differentiate virulent *C. concisus* strains from commensal strains.

A recent study reported the identification of an exoprotein named *C. concisus* secreted protein 1 (Csep1) (Liu et al., 2018). The *csep1* gene was found to be localized in the bacterial chromosome and the pICON plasmid. The chromosomally encoded *csep1* gene was only found in GS2 *C. concisus* strains, while the pICON plasmid encoded *csep1* gene was found in both GS1 and GS2 strains. Some *csep1* genes contained a six-nucleotide insertion at the position 654–659 bp (*csep1-6bpi*). Importantly, the *csep1-6bpi* gene in oral *C. consisus* strains was found to be associated with active CD. So far, the *csep1-6bpi* gene is the only molecular marker in *C. concisus* reported to have an association with IBD.

### Pathogenic mechanisms of *C. concisus*

#### Motility, adhesion, and invasion

*Campylobacter* movement through the mucus layer is driven by its polar flagellum, which is crucial for approaching, attaching, and invading the intestinal epithelial cells (Young et al., 2007). In *C. jejuni*, the extracellular flagella filament is composed of multimers of flagellin proteins with a major flagellin protein FlaA and a minor flagellin protein FlaB. FlaA has been shown to be essential for invasion of INT 407 cells and optimal colonization in chicken gut (Wassenaar et al., 1991, 1993). The flagellum also serves as a type III secretion system for transportation of *Campylobacter* invasion antigens to the host cells (Buelow et al., 2011; Neal-Mckinney and Konkel, 2012; Samuelson et al., 2013).

**Figure 3:**
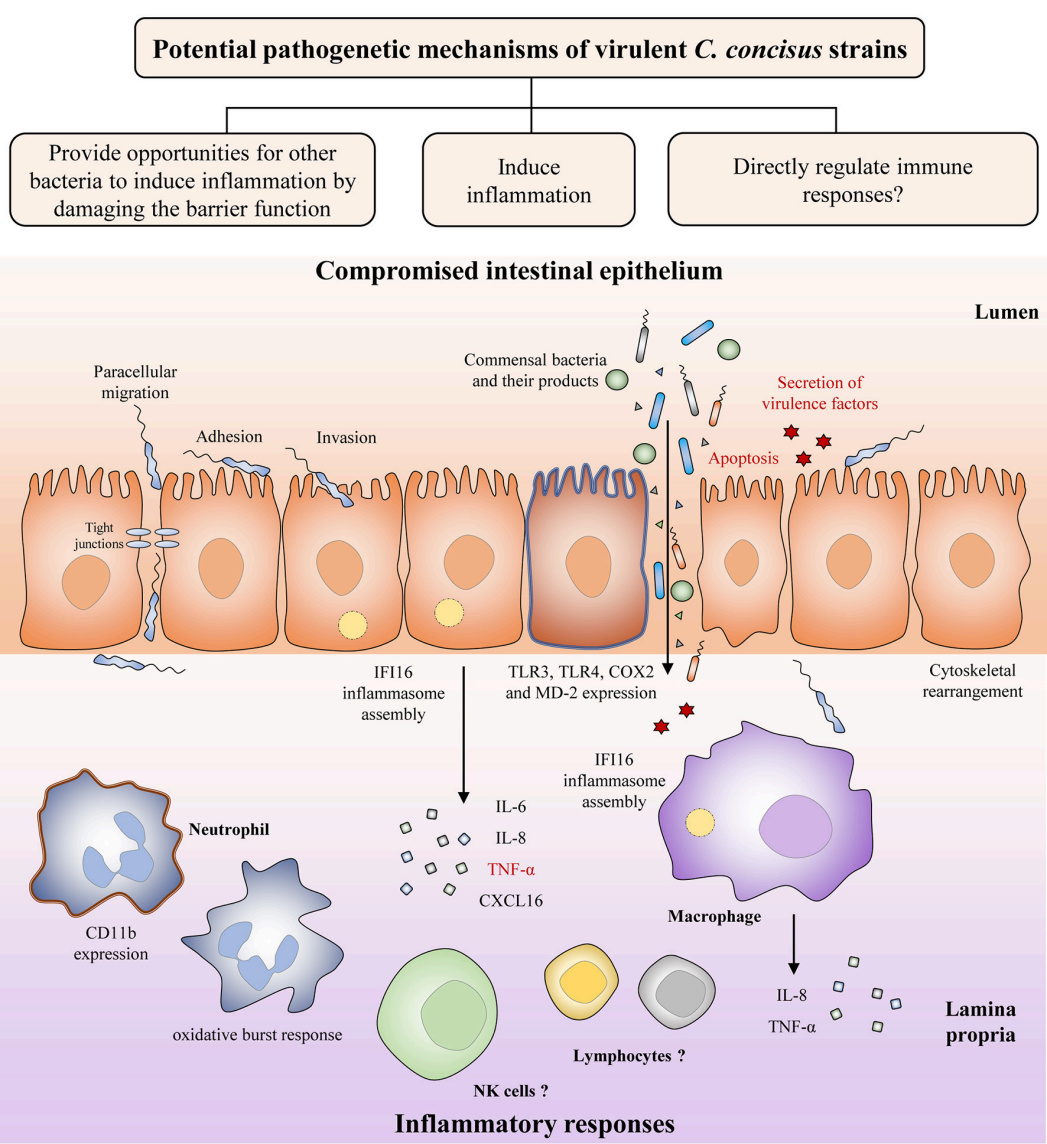
Potential pathogenetic mechanisms of virulent *C. concisus* strains. Once it reaches the intestine, *C. concisus* adheres and invades the epithelium with the help of its flagellin and spiral shape. This is followed by a number of host responses such as cytoskeletal rearrangement, inflammasome assembly, expression of toll-like receptors, and the release of proinflammatory cytokines. Expression of virulence factors such as the zonula occludens toxin also results in proinflammatory cytokine production, as well as apoptosis (colored in red). The resulting compromised intestinal epithelium allows the increased translocation of commensal bacteria and their products from the lumen to the lamina propria. Proinflammatory cytokines and chemokines released by intestinal epithelial cells recruit immune cells to the site of infection and contributes to inflammatory response development.

Both *C. concisus* and *C. jejuni* are spiral shaped. Unlike *C. jejuni*. which can have either single or bi-polar flagella, *C. concisus* only has a single polar flagellum. Although the flagellum in *C. concisus* has not been comprehensively investigated, studies have shown that *C. concisus* flagellum might be a virulence factor that contributes to its pathogenicity. Man et al. have observed flagellum mediated attachment and invasion of *C. concisus* to Caco-2 cells (Figure 3) (Man et al., 2010a).

Several studies have examined the motility of *C. concisus* strains isolated from saliva, feces and intestinal biopsies of patients with IBD, gastroenteritis, and healthy individuals. The motility was shown to be strain dependent, and no statistically significant association was found between patients and healthy controls (Lavrencic et al., 2012; Ovesen et al., 2017). The authors also reported that the motility of *C. concisus* was lower than that of *C. jejuni* and *C. fetus* (Ovesen et al., 2017). Furthermore, comparative genomic analysis of *C. concisus* strains had identified proteins required for flagellin glycosylation pathway, which may affect bacterial flagellar filament assembly, autoagglutination, adhesion and invasion (Kaakoush et al., 2011).

Bacterial flagella are also involved in forming biofilm, a bacterial interaction that is important for its survival in the host (Reeser et al., 2007; Svensson et al., 2014). *C. concisus* was also shown to have the ability to form biofilms, and this ability did not differ between the strains examined (Lavrencic et al., 2012).

#### Damaging the intestinal epithelial barrier

The intestinal epithelium is constructed by simple columnar epithelial cells (Clevers, 2013). Increased intestinal permeability is known to be associated with a number of chronic human diseases including IBD, being considered as a risk factor for its development (Hollander et al., 1986; Wyatt et al., 1993; Irvine and Marshall, 2000; D'Incà et al., 2006; Meddings, 2008). A compromised intestinal epithelial barrier may lead to loss of tolerance to the commensal enteric microbiota. Using *in vitro* cell culture models, several studies have indicated that *C. concisus* is able to increase the intestinal permeability. The intestinal epithelial permeability in Caco-2 cells was increased following *C. concisus* infection, and *C. concisus* also induced movement of tight junction proteins zonula occludens-1 and occludin from cell membrane into cytosol (Figure 3) (Man et al., 2010a). These changes in tight junction protein arrangement significantly increased the barrier permeability (Man et al., 2010a). These effects were also observed in HT-29/B6 intestinal epithelial cells, in which the cell monolayers infected with both oral and fecal *C. concisus* strains revealed epithelial barrier dysfunction (Nielsen et al., 2011).

#### Induction of proinflammatory cytokines

Inflammatory cytokines are produced in enteric infections (Figure 3). *C. concisus* strains were able to stimulate the productions of interleukin (IL)-8 and tumor necrosis factor (TNF)-α in THP-1 macrophages, and the productions of IL-8 and cyclooxygenase (COX)-2 in HT-29 cells (Man et al., 2010a; Ismail et al., 2013). COX-2 is an enzyme responsible for producing prostaglandins and other inflammatory mediators (Williams et al., 1999). Some *C. concisus* strains, mostly those isolated from patients with IBD, have been shown to upregulate surface expression of lipopolysaccharides (LPS) receptors including Toll-like receptor (TLR) 4 and myeloid differentiation factor 2 in HT-29 cells (Ismail et al., 2013). Activation of neutrophil adherence molecule CD11b and oxidative burst response have been shown in neutrophils following *C. concisus* infection (Sørensen et al., 2013). Additionally, by using transcriptomics analysis, assembly of IFI16 inflammasome has been observed in both intestinal epithelial cells and macrophages following *C. concisus* infection (Kaakoush et al., 2015; Deshpande et al., 2016). The IFI16 inflammasome plays important role in the innate immune responses, acting as a nuclear pathogen sensor that promotes caspase-1 activation (Xiao, 2015). Production of mature proinflammatory cytokines, IL-1β, and 1L-18, is caspase-1 dependent (Xiao, 2015). A recent study has found that *C. concisus* was able to elevate the mRNA expression of p53, TNF-α, and IL-18 in Barrett's cell lines, however these effects have not been demonstrated at protein level (Namin et al., 2015).

Flagellin from various Gram-negative and Gram-positive bacteria are capable of triggering nuclear factor kappa light chain enhancer of activated B cells (NF-κB) signaling responses in intestinal epithelial cells, where it is recognized by TLR5 expressed on the surface of the host cells (Eaves-Pyles et al., 2001; Gewirtz et al., 2001). The conserved region on flagellin that is recognized by TLR5 has been studied in *Salmonella typhimurium* (Smith et al., 2003). *Campylobacter* flagellin shares limited sequence similarity with that of *S. typhimurium*, which explains why it is a poor stimulator of TLR5 (Watson and Galán, 2005). Studies have demonstrated that *C. jejuni, C. coli*, and *C. concisus* have limited potential to activate TLR5 (De Zoete et al., 2010; Ismail et al., 2013).

To date, only two virulence factors of *C. concisus* have been characterized, one of which is the zonula occludens toxin (Zot). Initially described in *Vibrio cholerae*, Zot is a known virulence factor that causes an increase in intestinal permeability. The *zot* genes in *Campylobacter* species are encoded by prophages and are divided into two clusters, which encode Zot_CampyType_1_ and Zot_CampyType_2_, respectively (Zhang et al., 2014; Liu et al., 2016). Although the two types of Zot toxins share common motifs, their overall sequence identities vary greatly, particularly at the C-terminal compartment (Liu et al., 2016). Mahendran et al. have detected a similar prevalence of the cluster 1 *zot* gene in the oral *C. concisus* strains isolated from patients with IBD and healthy controls (Mahendran et al., 2013). Later on, Mahendran and colleagues found that the Zot_CampyType_1_ caused prolonged damage on Caco-2 monolayers (Mahendran et al., 2016). The damaging effect is different from that of *V. cholerae* Zot which induces transient and reversible damage to the intestinal epithelium (Fasano et al., 1991). This prolonged damaging effect induced by *C. concisus* Zot is at least partially due to the induction of cell apoptosis and/or intestinal epithelial cell production of proinflammatory cytokines such as TNF-α and IL-8 (Mahendran et al., 2016). Furthermore, pre-exposure to Zot causes increased phagocytosis of *E. coli* K12 by THP-1 macrophages, suggesting a possible role for *C. concisus* in enhancing responses of macrophages to other enteric bacterial species (Mahendran et al., 2016). Additionally, transcriptomics analysis showed that *C. concisus* Zot was able to upregulate the expression of TLR3, proinflammatory cytokines IL-6, IL-8, and chemokine CXCL16 (Deshpande et al., 2016). The *zot*-containing prophages are also found in a number of other human and animal hosted *Campylobacter* species including *C. ureolyticus, Campylobacter corcagiensis, C. gracilis, C. jejuni, Campylobacter hyointestinalis*, and *Campylobacter iguanorium*, however their pathogenic effects on human cells have not been examined (Liu et al., 2016).

In additional to Zot, another *C. concisus* virulence factor characterized is the membrane protein phospholipase A. Istivan et al. have examined the activity of haemolytic phospholipase A_2_ (PLA_2_) from *C. concisus* strains isolated from children with gastroenteritis and found that PLA_2_ activity is detected in strains from both GS. Membrane extracts containing PLA_2_ exhibited cytolytic effects on CHO cells, supporting the idea that *C. concisus* may have potential to cause tissue destruction related to intestinal inflammation (Istivan et al., 2004).

To date, only one study has examined the effects of *C. concisus* in animal model (Aabenhus et al., 2008). Mice displayed a significant loss of body weight between day 2 and day 5 following *C. concisus* inoculation via gastric route. Signs of inflammation in the gut were not consistently found, but micro abscesses were found in liver of infected animals. Additionally, infiltration of lymphocytes was also observed in the jejunum and colon of infected mice. However, the details of the *C. concisus* strains used were not provided.

#### Responses to environmental factors

Environmental factors present in the gastrointestinal tract such as bile and pH have been found to affect the growth of *C. concisus in vitro* (Ma et al., 2015). Reduction in *C. concisus* growth was observed with lower pH. The gastric pH values in patients with CD and UC range between 1.5–4.1 and 1.55–4.4, respectively, which are significantly higher than those in healthy individuals (0.95–2.6) (Press et al., 1998). Under laboratory conditions, *C. concisus* strains were unable to grow following exposure to pH 2 for 30 min, while only 20% of the strains were able to survive following exposure to pH 3.5 for 30 min, and exposure to pH 5 for 120 min had minor effects on *C. concisus* growth (Ma et al., 2015). The sensitivity of *C. concisus* toward low pH suggests that the acidic environment may prevent *C. concisus* from colonizing the stomach and intestinal tract. The less acidic gastric environment observed in patients with IBD may provide a better condition for *C. concisus* colonization (Press et al., 1998). Recently, Ma et al. reported that the derivatives of the food additive, fumaric acid, were able to enhance the growth of *C. concisus* strains (Ma et al., 2018). It was found that *C. concisus* strains showed the greatest increase in growth when cultured in media containing 0.4% of neutralized fumaric acid, neutralized monosodium fumarate and sodium fumarate. These results imply that removal of fumaric acid and its salts from diet of patients with IBD may help to improve their condition.

*Campylobacter* species have different ability in bile resistance. Some oral *C. concisus* strains are able to grow in the presence of 2% bile, suggesting they are able to survive in the enteric environment. Enteric *Campylobacter* species such as *C. jejuni* and *C. hominis* are bile resistant. Fox et al. have demonstrated that although significant growth inhibition was observed when *C. jejuni* was cultured in the presence of 2.5% bile, *C. jejuni* was still able to proliferate in cultures containing 5% bile (Fox et al., 2007). Lawson et al. showed that *C. hominis* strains isolated from stool samples were able to tolerate 2% bile (Lawson et al., 2001).

### Antimicrobial resistance of *C. concisus*

There has been an increase in antibiotic resistance in *Campylobacter* isolates from both humans and animals worldwide (Luangtongkum et al., 2009; Iovine, 2013). Among antimicrobial therapies for treatment of *Campylobacter* enteritis, fluoroquinolones, and macrolides are most commonly used, while tetracyclines are rarely used (Alfredson and Korolik, 2007). Intravenous aminoglycosides are sometimes used to treat serious bacteraemia and other *Campylobacter* induced systemic infections (Alfredson and Korolik, 2007). *Campylobacter* species resist antibiotics by several mechanisms such as modification or occupation of target sites thus preventing the binding of antibiotic compounds; efflux pump systems that reduce intracellular antibiotic concentrations; changes in bacterial membrane permeability that prevents entering of antibiotic compounds; and hydrolysis of antibiotic compounds (Alfredson and Korolik, 2007; Iovine, 2013). The resistance determinants in *C. jejuni* and *C. coli* and their mechanisms of action have been widely studied, however the resistance determinants in *C. concisus* have not been investigated in detail. It is thought that antibiotics resistance has not developed in *C. concisus* as it is susceptible to a variety of antibiotics, of which tetracycline, erythromycin, ciprofloxacin, and macrolides are most commonly reported (Johnson et al., 1986; Aabenhus et al., 2005; Vandenberg et al., 2006; Nielsen et al., 2013). Ciprofloxacin, along with other antibiotics, have been shown to be effective in treating certain phenotypes of CD (Sartor, 2004).

Antibiotics are used as primary or adjuvant treatment along with anti-inflammatory and immunosuppressive drugs for the treatment of IBD (Sartor, 2004). Antibiotics are used to selectively reduce tissue invasion, decrease luminal, and mucosal bacterial loads and their translocation (Sartor, 2004). Some patients with IBD are colonized with multiple *C. concisus* strains, and a significantly higher prevalence of multiple oral *C. concisus* strains in patients with active IBD than healthy controls has been reported (Mahendran et al., 2013). Furthermore, for IBD patients who are in remission, those without antibiotic treatment usually have a higher prevalence of multiple oral *C. concisus* strains than those who were receiving antibiotics, indicating antibiotics were able to alleviate but not eradicate *C. concisus* growth in the intestinal tract (Mahendran et al., 2013).

In addition to antibiotics, the antimicrobial potentials of immunomodulating and anti-inflammatory drugs used for IBD treatments have not been widely tested. Immunosuppressive drugs such as azathioprine (AZA) and mercaptopurine (MP) used in the treatment of IBD have been reported to exhibit inhibitory effect on the growth of *C. concisus* strains, with the effect of AZA being more potent than MP (Liu F. et al., 2017). In their use as immunosuppressive drugs, both AZA and MP are eventually metabolized to purine analogs that interfere with DNA synthesis in immune cells (Nielsen et al., 2001). However, bioinformatics analysis has not identified all the enzymes required for AZA and MP metabolism in the *C. concisus* genome, indicating the inhibitory action of AZA and MP on *C. concisus* growth is not through the conventional pathway. AZA and MP can also reduce the growth of *Mycobacterium avium* subsp. *paratuberculosis*, another bacterium that is associated with human CD (Sanderson et al., 1992; Collins et al., 2000; Sieswerda and Bannatyne, 2006; Greenstein et al., 2007; Shin and Collins, 2008).

5-aminosalicylic acid (5-ASA) is an anti-inflammatory drug that is also commonly used to induce and maintain remission in IBD (Hanauer, 2006; Nikfar et al., 2009). Its effect on *C. concisus* growth varies between strains as it inhibits the growth of some *C. concisus* strains, while it promotes the growth of other strains, features which appears to be independent of *C. concisus* GS (Schwartz et al., 1982). Although 5-ASA is often used in the treatment of IBD, in some cases 5-ASA medications are found to cause exacerbations of colitis (Schwartz et al., 1982). The enhancement of *C. concisus* growth induced by 5-ASA may have implications in the deteriorated clinical conditions observed in patients with IBD following 5-ASA medications.

While antimicrobials may be effective in limiting the growth of *C. concisus* in the intestinal tract, continuous transportation of *C. concisus* from the oral cavity along with saliva and food makes eradication of this bacterium a challenge. Thus, antimicrobials targeting *C. concisus* in the oral cavity, particularly virulent strains, should be developed.

## C. rectus

*C. rectus* (formerly named as *Wolinella recta*) is a small, straight, rod shaped, single polar flagellated bacterium with the size being 0.5 by 2–4 μm (Tanner et al., 1981). The colonies of *C. rectus* appear as convex shaped and spread or corrode blood agar plates (Tanner et al., 1981). *C. rectus* was initially identified using anaerobic condition consisting of 80% N_2_, 10% CO_2_, and 10% H_2_, although the study indicated that some stains can grow in the presence of 5% oxygen (Tanner et al., 1981). A number of studies have used anaerobic conditions for the isolation of *C. rectus* from different clinical samples such as dental plaques, feces, and oesophageal mucosal biopsies (Rams et al., 1993; Gmur and Guggenheim, 1994; Von Troil-Lindén et al., 1995; Lastovica and Roux, 2000; Macuch and Tanner, 2000; Macfarlane et al., 2007). A study comparing different atmospheric conditions on the growth of *C. rectus* demonstrated that its growth could be observed at 30, 35, and 42°C under anaerobic conditions; in contrast it did not grow in a conventional microaerophilic atmosphere consisting 5% O_2_, 10% CO_2_, and 85% N_2_ (Mahlen and Clarridge, 2009). Given all these findings, *C. rectus* is an anaerobic bacterium instead of microaerophilic bacterium.

*C. rectus* was first isolated from individuals with periodontal diseases (Tanner et al., 1981). It has since been isolated from various locations of the oral cavity including periodontal sulcus, tongue, cheek mucosa, and saliva (Könönen et al., 2007; Cortelli et al., 2008). *C. rectus* is predominantly localized in the middle and deep periodontal pocket zones, and tends to form clumps in tooth-attached and epithelium associated plaque areas (Noiri et al., 1997). A number of studies have reported the association between *C. rectus* and periodontal diseases. Macuch and Tanner detected *C. rectus* from 90% (42/47) of the subgingival sites of initial and established perondontitis individuals, which was signficianlty higher than that from gingivitis subjects (20%, 3/14) and healthy controls (10%, 2/18) (Macuch and Tanner, 2000). Dibart et al. also showed that *C. rectus* had a signfiicantly higher prevalence in supragingival plaque obtained from periodontally diseased subjects as comapred with healthy individuals (7/24 vs. 0/27) (Dibart et al., 1998). Furthermore, von Troil-Lindenl et al. reported that *C. rectus* was more frequently detected in saliva samples of subjects with advanced periondontitis (100%, 10/10) as comapred with individuals with initial or no periondontitis (40%, 4/10) (Von Troil-Lindén et al., 1995). Additionally, several studies have examined the prevalence of *C. rectus* in periondontitis patients with different disease status, however healthy subjects were not included. Several virulence factors of *C. rectus* including LPS, GroEL-like protein, and surface-layer protein have been characterized.

*C. rectus* acts as a stimulator of inflammation in the gingival tissue. It has been shown to enhance the production of IL-6 and IL-8 in human gingival fibroblasts (Dongari-Bagtzoglou and Ebersole, 1996). IL-6 is a multifunctional cytokine, as it can be both proinflammatory and anti-inflammatory, and its secretion is thought to drive the tissue damaging process in diseases such as periodontal disease (Irwin and Myrillas, 1998). *C. rectus* LPS is able to stimulate both plasmin activity and plasminogen activator activity in human gingival fibroblasts (Ogura et al., 1995). Plasmin is an enzyme that is converted from plasminogen via plasminogen activator catalysis (Martel-Pelletier et al., 1991). Plasmin is present in blood which degrades many blood plasma proteins such as fibrin clots (Martel-Pelletier et al., 1991). Significantly enhanced activities of plasmin and plasminogen activator activities have been observed in gingival fluid in periodontal disease (Hidaka et al., 1981; Talonpoika et al., 1990). Furthermore, *C. rectus* LPS also induces higher levels of IL-6 and prostaglandin E2 productions in aged human gingival fibroblasts as compared with those in younger cells, which may explain the increased susceptibility of periodontal disease in aged individuals (Ogura et al., 1996; Takiguchi et al., 1996, 1997).

The *C. rectus* GroEL-like protein is a 64 kDa protein with antigenic properties (Hinode et al., 1998). It was found to cross-react with antibodies against human heat shock protein 60, *Helicobacter pylori* whole cells and the GroEL-like protein from *Actinobacillus actinomycetemcomitans*, a bacterial species that is associated with localized aggressive periodontitis (Hinode et al., 1998, 2002; Tanabe et al., 2003; Henderson et al., 2010). The *C. rectus* GroEL-like protein is also able to stimulate the production of IL-6 and IL-8 from the human gingival fibroblast monolayer (Hinode et al., 1998). The *C. rectus* GroEL-like protein is also found to possess immunodominant epitopes within both amino and carboxyl termini, and it may share same carboxyl epitopes with the *C. rectus* surface-layer (S-layer) protein (Hinode et al., 2002).

The S-layer is a monomolecular layer of a single secreted protein surrounding the entire surface which is found in almost all archaea and some Gram-positive and Gram-negative bacteria (Konstantinov et al., 2008). The S-layer protein (SLP) in *C. fetus* and *C. rectus* have been previously characterized, and the gene encoding SLP is also carried by *C. showae* (Tay et al., 2013). The *C. rectus* SLP has a molecular weight ranging between 130 and 166 kDa (Kobayashi et al., 1993; Nitta et al., 1997). *C. rectus* strains expressing SLP are resistant to complement and phagocytic mediated killing in the absence of specific antibodies (Okuda et al., 1997). However, *C. rectus* SLP does not play a major role in bacterial adhesion. *C. rectus* strains lacking the *crsA* gene which encodes the SLP protein are less effective at adhering to Hep-2 oral epithelial cells, with CrsA+ cells only 30 to 50% more adherent than the CrsA- cells (Wang et al., 2000). The CrsA- cells exhibit no difference in inducing the production of IL-6, IL-8, and TNF-α in Hep-2 cells as compared with CrsA+ cells. However, CrsA- *C. rectus* strains induce higher levels of these cytokines at early time points of the infection, suggesting that the S-layer in *C. rectus* may facilitate bacterial survival at the site of infection by delaying the cytokine responses (Wang et al., 2000).

Macrolides are a class of antibiotics used to treat a wide variety of infections (Hirsch et al., 2012). It inhibits protein synthesis by acting on the P site of the 50S ribosomal subunit, thus preventing peptide elongation (Payot et al., 2006). The rRNA methylase enzyme encoded by the erythromycin ribosome methylase (*erm*) gene methylates a single adenine in the 23S component of the 50S subunit, which sterically hinders the proper interaction between the macrolide and the 50S subunit, providing antibiotic resistance (Weisblum, 1995). *C. rectus* is the only *Campylobacter* species that has the *erm* determinants described. These determinants include Erm B, Erm C, Erm F, and Erm Q, though their clinical implications have not been examined (Roe et al., 1995).

In addition to its role in periodontal disease, *C. rectus* has been implicated in the association between maternal periodontitis and adverse pregnancy outcomes. Madianos et al. reported that a significantly elevated level of fetal IgM to *C. rectus* was observed among premature infants as compared to full-term neonates, indicating that *C. rectus*, as a maternal oral pathogen, may act as a primary infectious agent in fetus that leads to prematurity (Madianos et al., 2001). Later study by the same group demonstrated that *C. rectus* mediated growth restriction in a pregnant mice model, as higher numbers of growth-restricted fetuses were observed in the groups subcutaneously challenged by *C. rectus* as compared to the non-challenged groups (Yeo et al., 2005). The same group also showed that maternal *C. rectus* infection increased fetal brain expression of proinflammatory cytokines IFN-γ (Offenbacher et al., 2005). Furthermore, Arce and colleagues demonstrated that *C. rectus* was more invasive to human trophoblasts as compared to *C. jejuni*, paralleled with significantly upregulated mRNA and protein expressions of IL-6 and TNF-α in a dose-dependent manner (Arce et al., 2010). Moreover, the study also showed that *C. rectus* was able to translocate *in vivo* from a distant site of infection to the fetoplacental unit (Arce et al., 2010). Taking together, these studies suggest that as a pathogen in the oral cavity, *C. rectus* has the potential to contribute to the adverse pregnancy outcomes.

## C. ureolyticus

*C. ureolyticus* (formally known as *Bacteroides ureolyticus*) was first described in 1978 by Jackson et al., the strain was isolated from amniotic fluid using anaerobic condition (Jackson and Goodman, 1978). A later study has isolated *C. ureolyticus* from 103 superficial necrotic or gangrenous lesions. However, *C. ureolyticus* was rarely the sole bacterial species isolated from the site of infections, suggesting other microorganisms may be responsible for the pathogenesis of these diseases (Duerden et al., 1982).

*C. ureolyticus* has been frequently isolated from the genital tracts of both male and female. By examining the whole cell proteins of *C. ureolyticus* strains, Akhtar and Eley showed that strains isolated from urethra of men with and without non-gonococcal urethritis showed no difference in SDS-PAGE patterns (Akhtar and Eley, 1992). Later studies by Bennett et al. reported that *C. ureolyticus* was isolated from healthy individuals at a similar prevalence as compared with that from males and females presenting non-gonococcal urethritis (Bennett et al., 1990, 1991). These results indicate that *C. ureolyticus* is a part of the normal flora in the genital tract of both male and female.

In the past decades, *C. ureolyticus* has been considered as an emergent *Campylobacter* species in gastroenteritis as it is frequently isolated from fecal samples of patients presenting with diarrheal illness. By using a multiplex-PCR systems, Bullman et al. first reported the isolation of *C. ureolyticus* from feces of patients with gastroenteritis. Among all the *Campylobacter* positive samples being screened, 24% (83/349) was found to be positive for *C. ureolyticus*, of which 64% (53/83) had *C. ureolycitus* as the sole *Campylobacter* species detected (Bullman et al., 2011). By using PCR methods, another study by Bullman et al. also found that *C. ureolyticus* was the second most common non-*C. jejuni*/*C. coli* species in fecal samples collected from patients presenting diarrheal illness (Bullman et al., 2012). However, healthy controls were not included in these studies.

By combining the bacterial isolation and molecular detection methods, Collado et al. reported that *C. ureolyticus* is the third most prevalent species of the Campylobacteria family in diarrheic stool samples in addition to *C. concisus* and *C. jejuni*, but no statistical difference was found between the diarrheic and healthy groups (Collado et al., 2013). A recent study has also reported the detection of *C. ureolyticus* from traveler's diarrhea cases (Serichantalergs et al., 2017). This study showed that *C. ureolyticus* was detected at a similar prevalence in patients presenting diarrhea and healthy individuals.

Despite being frequently isolated or detected from diarrheal stool samples, the association between *C. ureolyticus* and diarrheal diseases has not been established. However, the pathogenic potentials of *C. ureolyticus* have been examined by a number of *in vitro* studies. Through whole genome sequencing of *C. ureolyticus* strains, genes encoding known virulence factors have been identified. These factors were involved in bacterial adhesion, colonization, invasion, and toxin production (Bullman et al., 2013). By using an intestinal epithelial cell line model, *C. ureolyticus* was shown to be capable of adhering to Caco-2 cells but unable to invade, with the bacterial adhesion followed by cellular damage and microvillus degradation. Furthermore, secretome analysis had also detected release of putative virulence and colonization factors (Burgos-Portugal et al., 2012).

Hariharan et al. reported the isolation of *C. ureolyticus* from endometria of apparently normal mares under anaerobic condition (Hariharan et al., 1994). So far, this is the only study has reported isolation of *C. ureolyticus* from animal sources.

## C. gracilis

*C. gracilis* (formally known as *Bacteroides gracilis*) was first isolated by Tanner et al. in 1981 from patients with gingivitis and periodontitis (Tanner et al., 1981).

*C. gracilis* has been isolated from patients with periodontal and endodontic infections, however its pathogenetic role in these diseases is still controversial. Macuch et al. reported that in addition to *C. rectus, C. gracilis* was the most dominant *Campylobacter* species isolated from subgingival sites among eight *Campylobacter* species being examined. However, similar levels of *C. gracilis* were detected in healthy, gingivitis and periodontitis sites, suggesting that its prevalence or quantity was unrelated to periodontal health or disease (Macuch and Tanner, 2000). A study by Siqueira and Rocas examining the prevalence of *C. gracilis* in patients with primary endodontic infections also found that it was not associated with clinical symptoms of the diseases (Siqueira and Rocas, 2003).

The first complete genome of *C. gracilis* was sequenced by Miller et al., which lead to identification of genes encoding for virulence factors including haemagglutinins, Zot, immunity proteins and other putative pathogenic factors (Miller and Yee, 2015). Nevertheless, supporting evidence for the virulence of *C. gracilis* is still lacking.

## C. showae, C. hominis, and C. curvus

*C. showae* was first isolated by Etoh et al. in 1993 from dental plaque of gingival crevices of healthy adults (Etoh et al., 1993). A study later by Macuch et al. examining the prevalence of oral *Campylobacter* species from subgingival sites showed that in addition to *C. rectus, C. showae* was also found more frequently and in higher levels from patients with periodontitis and gingivitis than from healthy controls (Macuch and Tanner, 2000).

*C. hominis* was first described in 1998 by Lawson et al. through *Campylobacter* specific PCR assays. *C. hominis* was detected from stool samples of 50% of the 20 healthy individuals, while it was absent in all the saliva samples of the same individuals (Lawson et al., 1998). Later in a study by Lawson et al., *C. hominis* was isolated from the fecal samples of healthy individuals (Lawson et al., 2001).

By using PCR methods targeting the *Campylobacter* 16S rRNA gene, *C. showae* and *C. hominis* have been detected from intestinal biopsy samples collected from macroscopically inflamed and noninflamed areas. Furthermore, this was the first study reported the isolation of *C. concisus, C. showae, C ureolyticus*, and *C. hominis* from intestinal biopsies (Zhang et al., 2009). A later study by Man et al. also detected *C. showae* and *C. hominis* from stool specimens collected from children with CD (Man et al., 2010b). However, no association was found between the prevalence of *C. showae* and *C. hominis* in patients with CD and healthy controls.

*C. curvus* (previously named as *Wolinella curva*) was first described in 1984 by Tanner et al. (Tanner et al., 1984). *C. curvus* is a rarely encountered *Campylobacter* species in humans. Lastovica et al. reported an isolation rate of *C. curvus* as low as 0.05% (2/4122) from diarrheic stools of pediatric patients (Lastovica and Roux, 2000). A study by Abbott et al. also isolated *C curvus* from stool samples and showed that the prevalence of *C. curvus* was associated with sporadic and outbreak of bloody gastroenteritis and Brainerd's diarrhea in Northern California (Abbott et al., 2005). There was one study which reported isolation of *C. curvus* and *C. upsaliensis* from stools of patients with Guillain-Barré syndrome and Fisher's syndrome (Koga et al., 1999). However, serological examination of the patient from whom *C. curvus* has been isolated did not detect antibodies to this bacterium.

## Conclusion

Accumulated evidence from the past decade supports the role of human hosted *C. concisus* in the development of IBD and possibly Barrett's esophagus and GERD. Recently, a CD-associated *C. concisus* molecular marker *csep1-6bpi* has been identified, strongly suggests that *csep1-6bpi* positive *C. concisus* strains is a potential causative agent of human CD. In addition to *C. concisus*, the association between human hosted *C. rectus* and periodontal diseases has also been established.

## Author contributions

FL played the major role in writing the review. LZ, RM, and YW provided critical feedback and helped in editing the manuscript.

### Conflict of interest statement

The authors declare that the research was conducted in the absence of any commercial or financial relationships that could be construed as a potential conflict of interest.

## Abbreviations

5-ASA: 5-aminosalicylic acid
AZA: azathioprine
CD: Crohn's disease
CFU: colony forming unit
COX: cyclooxygenase
Erm: erythromycin ribosome methylase
GERD: gastroesophageal reflux disease
GS: genomospecies
IBD: inflammatory bowel disease
IFN: interferon
IL: interleukin
LPS: lipopolysaccharide
MP: mercaptopurine
NF-κB: nuclear factor kappa-light-chain-enhancer of activated B cells
PCR: polymerase chain reaction
PLA_2_: phospholipase A_2_
TLR: toll-like receptor
TNF: tumor necrosis factor
UC: ulcerative colitis
Zot: zonula occludes toxin.
